# Mechanical properties of ECC connection plates in seamless Bridges

**DOI:** 10.1371/journal.pone.0351751

**Published:** 2026-06-17

**Authors:** Ziwang Xiao, Lifeng Wang, Rikang Huang

**Affiliations:** School of Civil Engineering and Transportation, Northeast Forestry University, Harbin, China; SRM Institute of Science and Technology: SRM Institute of Science and Technology (Deemed to be University), INDIA

## Abstract

Seamless bridges require high-performance connection systems due to the elimination of expansion devices. This study investigates the flexural performance of ECC connection plates (ECP) for seamless RC bridge decks using finite element analysis. A three-dimensional ABAQUS model was developed and validated against available experimental results from the literature. Parametric analyses were performed to evaluate the effects of ECC thickness, reinforcement ratio, concrete strength, and ECC strength. The results show that ECP significantly improves flexural capacity and ductility compared with conventional RC connection plates. Under the same reinforcement ratio, the ultimate load and ductility coefficient increased by 35.1% and 31.3%, respectively. Increasing ECC thickness and ECC strength enhances both load-bearing capacity and deformation ability, while a higher reinforcement ratio increases ultimate load but reduces tensile strain capacity. A theoretical formula for predicting the flexural bearing capacity of ECP was derived and verified using numerical results. The findings provide a basis for the design and optimization of ECP in seamless bridge decks.

## 1. Introduction

In the field of bridge engineering, the installation of bridge deck expansion joints represents a conventional approach to balancing structural deformation adaptability with durability. This design aims to mitigate deformation effects between adjacent bridge spans caused by external loads and temperature variations through appropriate construction measures, while simultaneously blocking water seepage paths to prevent corrosion of the main girders [1,2]. However, this construction method has revealed numerous issues in practical applications: the infiltration of chloride-laden water not only accelerates the aging and failure of the expansion joint components, thereby shortening their service life, but also compromises the durability of the main structural elements. Moreover, the discontinuity of the main girder at the expansion joint reduces ride comfort and significantly affects the operational performance of medium and small-span bridges [3–5]. Ma et al. [6] found that bridge expansion joints are directly subjected to repeated dynamic loads, making them susceptible to fatigue damage and necessitating traffic interruptions during replacement, which seriously affects the bridge’s normal operation. Hou et al. [7] investigated the dynamic response of bridges under vehicle loading and observed that expansion joint components are prone to fatigue failure under the combined effects of multiple structural defects and repeated heavy vehicle loads. Busel et al. [8] further noted that damage to expansion joint devices under various loading conditions significantly reduces traffic comfort, particularly at road intersections. Lima et al. [9] conducted the first comprehensive survey of 150 bridge expansion joints and reported that the maintenance requirements for these components were significantly higher than those for other bridge elements, resulting in disproportionately high maintenance costs. Li et al. [10] performed internal vibration tests on long-span bridges in different environmental conditions and found that pavement flatness was the primary factor influencing vertical vehicle vibration, with the maximum vibration amplitude typically occurring at the expansion joint locations.

**Fig 1:**
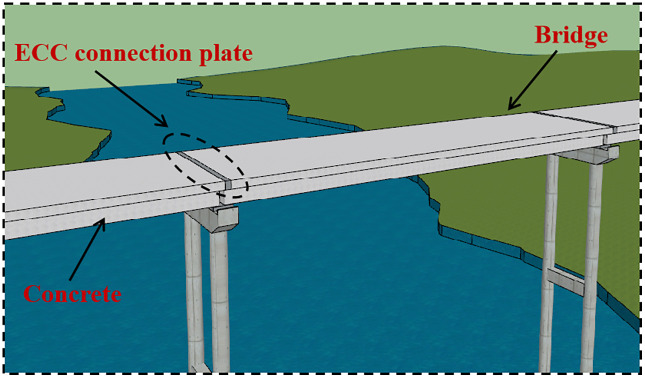
Application of ECP in Bridge Engineering.

In light of these challenges, the elimination of expansion joints has emerged as a significant research direction aimed at enhancing the performance of medium- and small-span bridges. Zuk [11] was the first to conduct foundational research on expansion joint-free bridge systems. Wasserman et al. [12] further refined the technical design of construction details for jointless beam bridges, establishing a theoretical basis for the engineering application of this structural concept. However, in bridges without expansion joints, due to the inherent characteristics of conventional materials, RC structures are susceptible to micro-cracking under minor tensile stresses. These micro-cracks tend to propagate into wider cracks under sustained loading and environmental exposure, leading to severe durability issues [13,14]. Engineered cementitious composites (ECC), as a novel class of high-ductility and high-toughness construction materials, exhibit pronounced strain-hardening behavior under tension. They can maintain substantial load-bearing capacity even after cracking, while effectively controlling crack width—typically within 100 μm—thus preventing the ingress of deleterious substances [15–17]. Additionally, ECC demonstrates excellent fatigue resistance and self-healing capabilities, and is less prone to brittle failure under cyclic loading, offering distinct advantages in enhancing structural durability [18,19]. In light of these properties, this study proposes the use of ECC as an alternative to conventional concrete for bridge deck connection plates—referred to as ECC connection plates (ECP)—to improve the mechanical performance, ride comfort, and durability of bridges. This approach demonstrates promising application potential in bridge engineering, as illustrated in Fig 1.

In ECP structures, the use of ECC is characterized by a relatively low elastic modulus, which allows for greater tensile and compressive deformation capacity. Through rational dimensioning of ECP elements, expansion joint devices can be effectively eliminated, resulting in a continuous bridge deck and significantly improved ride comfort. Furthermore, ECP exhibits a longer service life and maintenance interval compared to conventional RC connection plates, making it an ideal alternative to rigid bridge deck continuous systems. In addition, although ECC generally has a higher cement content than conventional concrete, its superior durability, crack control capability, and extended service life can significantly reduce repair frequency and material consumption over the life cycle of bridge structures, thereby mitigating its overall environmental impact and contributing to sustainable infrastructure development [20]. The superior durability and fatigue resistance of ECC allow it to withstand long-term cyclic wheel loads, a capability supported by extensive research. Ge et al. [21] conducted bending tests on ECC-concrete composite beams with varying ECC replacement ratios based on simplified ECC constitutive models. The results demonstrated that, compared to conventional RC beams, ECC-concrete composite beams exhibited significantly enhanced flexural capacity and ductility. Additionally, crack widths prior to rabar yielding were markedly reduced, and the ultimate curvature at tensile failure increased with higher ECC replacement ratios. Leung et al. [22] found that increasing the thickness of the ECC layer applied to the tension side of RC beams led to improved flexural capacity. Deng et al. [23] reported that increasing the fiber content in ECC enhanced its compressive strength, enabling the material to continue bearing loads after cracking and exhibiting pronounced strain-hardening behavior.

In practical applications and comparative studies, F. Castal [24] introduced the concept and design methodology of concrete connection plates to evaluate the performance of multi-span seamless bridges using traditional RC connection plates versus ECP. The results demonstrated that ECP exhibited superior performance compared to conventional concrete connection plates. Chu et al. [25] investigated the fatigue behavior of ECP after 400,000 loading cycles at stress levels of 40% and 55%, confirming that ECC outperformed ordinary concrete and maintained higher residual strength. ECC has been applied in bridge deck construction in several engineering projects. In 2002, ECC was used for localized bridge deck repairs [26], where the repaired sections exhibited excellent durability with no further micro-crack development. In Japan, ECC has been employed in steel bridge pavements [27], where its high toughness and crack self-healing properties effectively meet the durability and serviceability requirements of bridge decks. Additionally, the partial use of ECC resulted in a nearly 40% reduction in deck weight. Furthermore, Zhang et al. [28] investigated the mechanical performance and self-healing mechanisms of ECC, simulating the response of ECC bridge decks on steel girders under vehicular loading. Their findings confirmed that ECC performed well under fatigue loading conditions. To evaluate the crack propagation behavior of ECC, Zhang et al. [29] conducted tensile and flexural tests on two ECC composite beams, observing minimal crack widths and no water permeation, thereby ensuring structural durability. Gabriel et al. [30] assessed the performance of ECC with low fiber content and found that the critical tensile stress at the bottom of the pavement was significantly reduced, which not only mitigated brittle failure in rigid pavements but also allowed for a reduction in pavement thickness. Hou et al. [31] studied the effect of ECC on the fatigue performance of seamless bridge deck connection plates and found that ECC effectively reduced the strain fluctuation range of rebar, enhanced the energy dissipation capacity of specimens, and consequently extended the fatigue life of the rebar. Okeil and Adel [32] proposed an analytical approach for seamless bridges and validated its effectiveness through finite element modeling. Based on the above, ECC exhibits outstanding ductility, strain-hardening properties, and crack control capability, demonstrating significant potential for extending bridge fatigue life and meeting the requirements of structures subjected to long-term cyclic loading.

**Fig 2:**
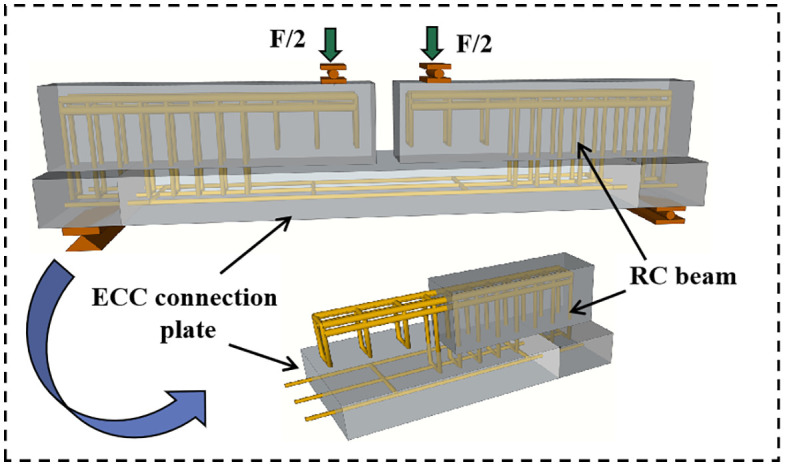
Schematic diagram of ECP construction in a seamless beam bridge.

Despite these advantages, a comprehensive review of existing literature indicates that research specifically on ECPs for seamless bridge decks remains limited, and the influence of key design parameters on their mechanical performance is still not fully understood. To address this research gap, this study aims to systematically investigate the behavior of ECPs under bending loads, highlighting both practical and theoretical implications. The ECP structure of the seamless beam bridge is shown in Fig 2. A three-dimensional finite element model of the ECP was developed using ABAQUS, and the simulated failure modes and load–deflection curves were compared with experimental results reported in the literature [25], validating the reliability of the model. Using this validated model, key parameters—including ECP thickness, reinforcement ratio, concrete strength, and ECC strength—were systematically analyzed to quantify their effects on structural performance. Furthermore, a theoretical formula for predicting the flexural bearing capacity of ECPs was derived, providing a practical tool for engineering design. The findings of this study not only fill a knowledge gap in the design of ECC connection plates but also offer guidance for optimizing the mechanical performance of seamless bridge decks, thereby contributing to both research and practical applications.

## 2. Method

### 2.1. Overview of the experiment

**Fig 3:**
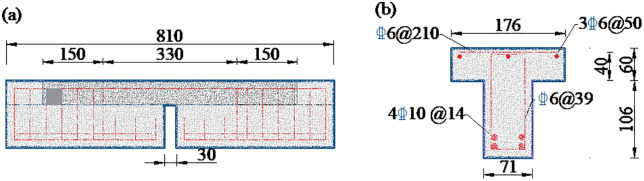
Schematic diagram of dimensions and rebar of the experimental beam(a) Longitudinal section; (b) Cross-section (unit: mm).

The dimensions and rebar details of the ECP in the RC seamless beam bridge investigated in this study are illustrated in Fig 3, and the corresponding basic parameters are summarized in Table 1. The total length of the ECP is 810 mm, with a 30 mm gap maintained between the RC beams to accommodate rotational deformation and displacement, as depicted in Fig 3(a). Fig 3(b) presents the T-shaped web, which has cross-sectional dimensions of 71 mm × 106 mm. It contains four longitudinal rebar with a diameter of 10 mm, and stirrups with a diameter of 6 mm spaced at 39 mm. In the transition zone where the web extends to the top plate, the stirrup spacing is reduced to 20 mm to enhance local structural integrity. The ECP is connected to the adjacent bridge deck using three 6 mm diameter rebar. The top plate has a cross-sectional dimension of 176 mm × 60 mm, with an ECC segment length of 330 mm and a total transition zone length of 630 mm. Laterally, 6 mm diameter rebar are arranged at a spacing of 210 mm within the ECP. At the material scale, the ECC matrix is reinforced with uniformly dispersed short fibers, which bridge microcracks and promote strain-hardening behavior through multiple fine cracking, thereby ensuring the ductile deformation capacity of the ECP [33]. During the experimental setup, the specimens were inverted so that the ECP was positioned in the tensile zone, ensuring structural stability during loading, as shown in Fig 2. The load applied to the top surface of the test beam simulated the reaction force exerted by the pier at the end of the adjacent beam [34]. For detailed design, implementation process, and result analysis of this experimental study, see reference [25].

**Table 1:** Characteristics of the test beam.

No.	ECC strength (MPa)	Longitudinal tensile rebar diameter (mm)	Concrete strength (MPa)	Thickness of ECP (mm)
LC-1	35	6	30	60

### 2.2. Finite element model

**Fig 4:**
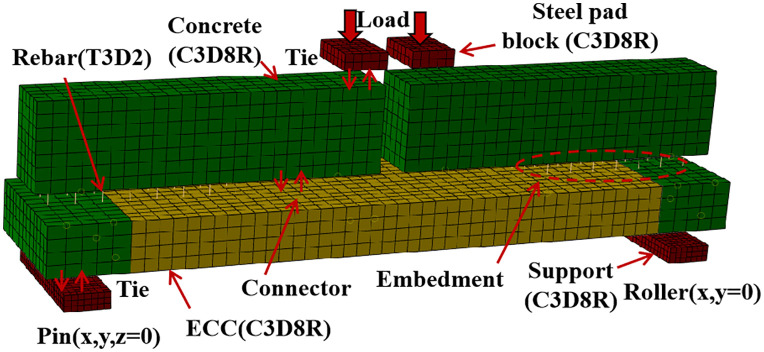
Finite element model of ECP.

The finite element model of the ECP was developed using ABAQUS software, under the assumption of perfect bond between the rebar and the surrounding concrete, with no slip considered [35]. To prevent stress concentration, rigid spacers were incorporated at both the support and loading points. In the finite element formulation, C3D8R solid elements were employed to simulate the concrete, spacers, and ECP. The rebar and stirrups were modeled using T3D2 three-dimensional truss elements. Connector elements were used to represent the interaction between the RC beams and the ECP, while rigid contact constraints were applied to prevent penetration between the concrete elements and the ECP under significant deformation [36]. The interface between the RC beam and the spacers was modeled using the “Tie” constraint method, and the rebar were embedded within the concrete using the “Embedded” constraint technique. The resulting finite element model of the ECP is illustrated in Fig 4, where a specified displacement was applied to the loading plate to ensure numerical convergence during the simulation.

A mesh sensitivity analysis was conducted using element sizes of 10, 15, 20, 30, and 50 mm for the C3D8R solid elements representing the ECC, concrete, and spacers. The resulting load–deflection curves and stress distributions were compared for each mesh size. It was observed that reducing the mesh size below 15 mm produced only marginal improvements in accuracy (less than 2% change in key response parameters), while significantly increasing computational cost. Conversely, coarser meshes (20 mm or larger) led to noticeable deviations in peak load and stress concentration. Based on this comparison, 15 mm was selected as the optimal mesh size, balancing accuracy and computational efficiency. The same mesh size and element types were used consistently for both the model validation and subsequent parametric analyses, ensuring the reliability and comparability of the results.

#### 2.2.1. Constitutive model of concrete.

**Fig 5:**
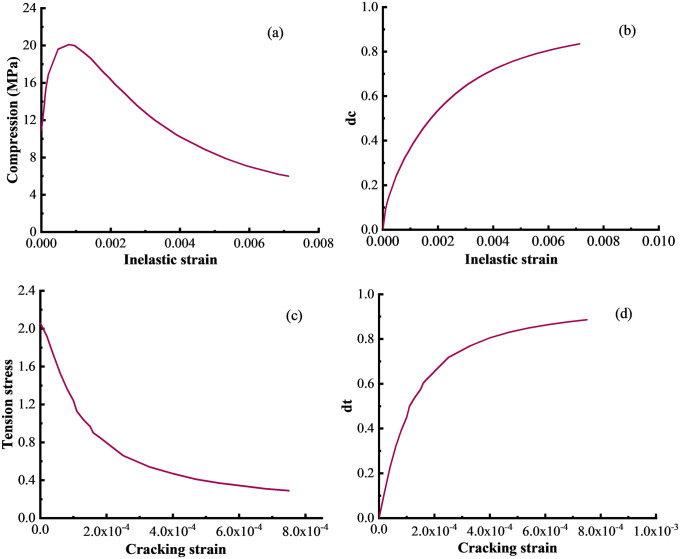
Constitutive relation curves of concrete: **(a)** Compressive stress vs. Inelastic strain; **(b)** Compressive damage vs. Inelastic strain; **(c)** Tensile stress vs. Cracking strain; **(d)** Tensile damage vs. Cracking strain.

The constitutive behavior of concrete was modeled using the Concrete Damaged Plasticity (CDP) model available in ABAQUS [37], which is capable of simulating cracking and failure mechanisms in concrete structures. The CDP model, based on the assumption of isotropic failure behavior, is applicable to unidirectional loading, cyclic loading, and dynamic loading conditions. It also accounts for the degradation of elastic stiffness due to tensile and compressive plastic strains, as well as the partial recovery of stiffness under cyclic loading, thereby ensuring good numerical convergence. The key parameters of the CDP model used in this study are summarized in Table 2. The concrete constitutive relationships were established in accordance with the current Chinese Code for Design of Concrete Structures (GB50010−2010) [38]. Fig 5 illustrates the stress-strain curves of the concrete employed in this study, along with the damage parameters under compression and tension. The mechanical properties of the concrete are listed in Table 3.

**Table 2:** CDP Model Parameters of concrete.

Parameter	Value
Expansion Angle	30 °
Eccentricity	0.1
*f*_b0_/*f*_c0_	1.16
K	0.667
Viscosity coefficient	0.001

**Table 3:** Mechanical properties parameters of concrete.

Item	*f’*_*c*_ (MPa)	*f*_*t*_ (MPa)	*Ec*	μ
Value	33.1	2.7	34453.9	0.2

#### 2.2.2. Constitutive model of rebar.

**Fig 6:**
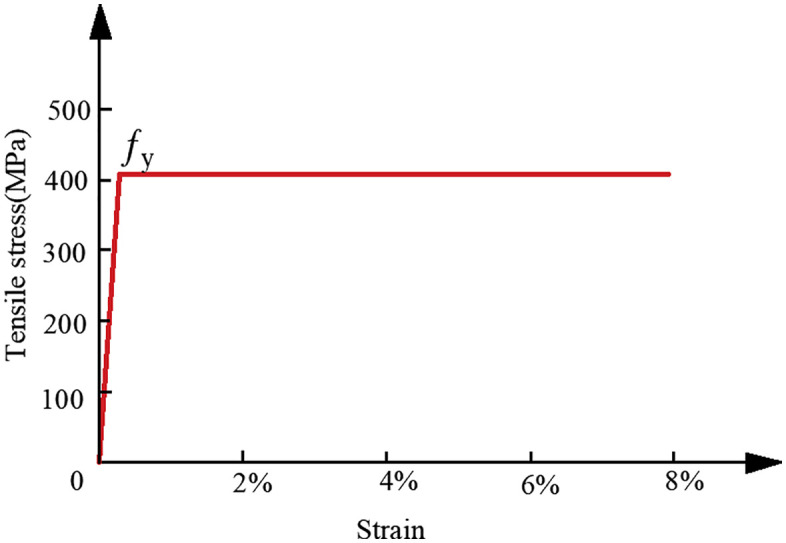
Constitutive curves of rebar.

In this study, an elastic-plastic material model was employed to simulate the longitudinal rebar and stirrups. The constitutive relationship incorporated both the elastic and plastic deformation stages, wherein the stress remained constant after reaching the yield strength, representing ideal plastic behavior [39]. The mechanical properties of the rebar are summarized in Table 4, and the corresponding stress-strain curves are presented in Fig 6.

**Table 4:** Mechanical Properties Parameters of rebar.

Rebar	Diameter (mm)	Elastic modulus (GPa)	Yield strength (MPa)	Poisson’s ratio
Tensile rebar	10	200	405	0.3
Compressed steel bars	6	200	405	0.3
Stirrups	6	200	300	0.3

#### 2.2.3. Constitutive model of ECC.

**Fig 7:**
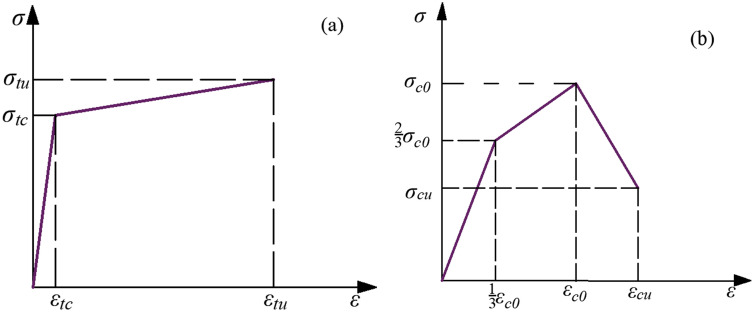
Stress-strain curve of ECC: (a) tensile;(b) Compression.


In recent years, scholars have carried out extensive studies on the constitutive modeling of ECC [40]. To simplify the computational process, the uniaxial tensile stress-strain behavior of ECC is approximated using a bilinear curve, as illustrated in Fig 7(a), which can be mathematically described by Equation (1). Similarly, the descending branch of the stress-strain curve under uniaxial compressive loading is also simplified to a bilinear form, as shown in Fig 7(b), and can be expressed by Equation (2).

$$\begin{array}{c} \sigma_{\text{t}}=\left\{ \begin{array}{c} @c@\frac{\sigma_{tc}}{\varepsilon_{tc}}\varepsilon \begin{array}{cc} & 0\leq \varepsilon <\varepsilon_{tc} \end{array}\\ \sigma_{tc}+\left(\sigma_{tu}-\sigma_{tc}\right)\left(\frac{\varepsilon -\varepsilon_{tc}}{\varepsilon_{tu}-\varepsilon_{tc}}\right)\begin{array}{cc} \begin{array}{cc} \begin{array}{cc} \begin{array}{c} \;\;\;\;\;\;\;\;\;\varepsilon_{tc}\leq \varepsilon \leq \varepsilon_{tu} \end{array} & \end{array} & \end{array} & \end{array} \end{array}\right. \end{array}$$ (1)

$$\begin{array}{c} \sigma_{c}=\left\{ \begin{array}{c} @c@2\frac{\sigma_{c0}}{\varepsilon_{c0}}\begin{array}{ccc} & & \begin{array}{cc} & \begin{array}{cc} & 0\leq \varepsilon <\frac{\text{1}}{\text{3}}\varepsilon_{c0} \end{array} \end{array} \end{array}\\ \frac{\text{2}}{\text{3}}\sigma_{c0}+\frac{\sigma_{c0}}{2\varepsilon_{c0}}\left(\varepsilon -\frac{\varepsilon_{c0}}{\text{3}}\right)\begin{array}{c} \;\;\;\;\;\;\;\;\frac{\text{1}}{\text{3}}\varepsilon_{c0}\leq \varepsilon <\varepsilon_{c0} \end{array}\\ \sigma_{c0}+\left(\sigma_{cu}-\sigma_{c0}\right)\left(\frac{\varepsilon -\varepsilon_{c0}}{\varepsilon_{cu}-\varepsilon_{c0}}\right)\begin{array}{c} \;\;\;\;\varepsilon_{c0}\leq \varepsilon <\varepsilon_{cu} \end{array} \end{array}\right. \end{array}$$ (2)

Among them, $E_{\text{0}}$ is the elastic modulus of ECC; $\varepsilon_{0.4}$ is the strain when the stress is 0.4 times the ultimate compressive strength in the stress rise section; $\varepsilon_{c0}$ is the strain at the ultimate compressive strength; $\sigma_{c0}$ is the ultimate compressive strength; $\varepsilon_{cu}$ is ultimate compressive strain; α is the reduction coefficient of the elastic modulus during the strain hardening stage, defined as, $\alpha =a\frac{\varepsilon E_{\text{0}}}{\sigma_{\text{c0}}}-b$, where *a* = 0.308 and *b* = 0.124 are constants obtained by fitting the experimental data.

#### 2.2.4. ECC and concrete bonding slip.

**Fig 8:**
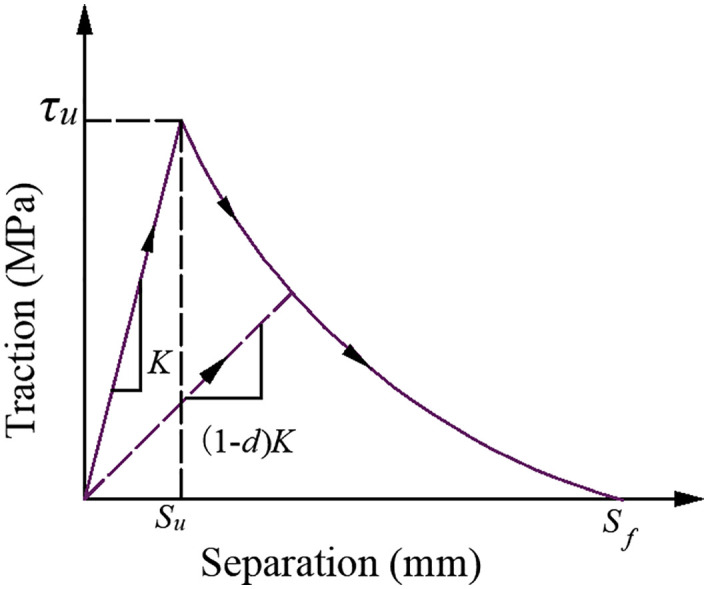
Bilinear bond-slip constitutive model.

To simulate the bonding slip between ECC and concrete, a zero-thickness bonding layer composed of COH3D8 elements was inserted between the ECP and the concrete interface, and the bonding layer was defined using the traction separation law [41]. The traction separation law is shown in Fig 8. The initial stage of the bilinear curve is set as linear elastic behavior, and when failure characteristics appear, the bilinear curve transforms into a damage process. The damage mechanism is the gradual degradation of the element stiffness [42]. Here, *K* represents the interfacial elastic stiffness, assuming that damage occurs when the slip reaches its limit $S_{u}$. $S_{f}$ is the final value after the descent. *d* is the damage variable.

### 2.3. Methods for determining ductility coefficients and yield loads

**Fig 9:**
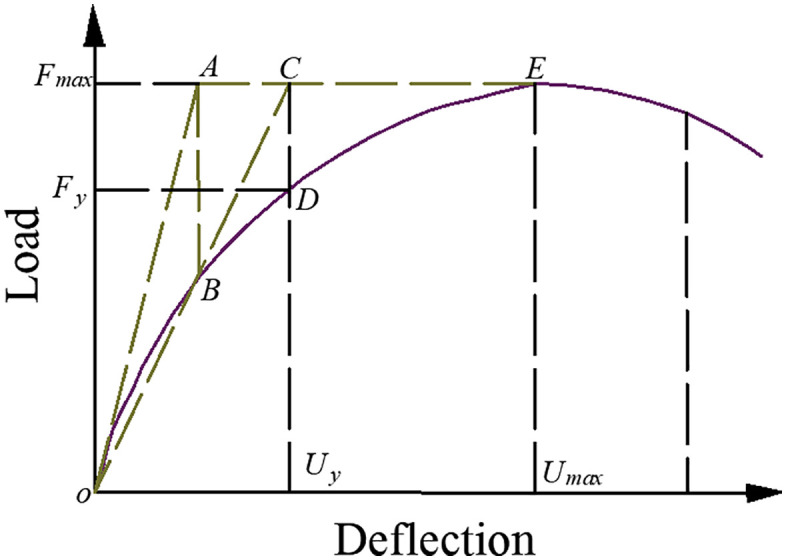
Schematic diagram of the yield moment method.


The ductility coefficient of the ECP was calculated using Equation (3) and analyzed accordingly [43]. In this study, the yield moment method was employed to determine the yield load [44], as illustrated in Fig 9. A tangent OA was drawn from the origin O of the coordinate system along the initial linear portion of the load-deflection curve, intersecting with the horizontal line AE drawn from the peak point A of the curve. A vertical line was then constructed through point A, intersecting the abscissa at point B. The extended line OB intersected line AE at point C. Subsequently, a vertical line CD was drawn from point C and intersected the load-deflection curve at point D. At this intersection, the horizontal coordinate of point D represents the yield deflection of the test beam, while the corresponding vertical coordinate indicates the yield load of the test beam.

$$\begin{array}{c} \mu =\frac{\Delta_{u}}{\Delta_{y}} \end{array}$$ (3)

Here, μ is the deflection ductility coefficient of the RC beam; Δ is the various parameters related to the deformation of the RC beam, such as deflection, strain, section curvature, rotation Angle, or deflection, etc., which are expressed as deflection in this study; $\Delta_{u}$ is the deflection corresponding to the ultimate load of the RC beam; $\Delta_{y}$ is the deflection corresponding to the yield load of the RC beam.

## 3. Results and discussion

### 3.1. Validation of the finite element model

#### 3.1.1. Load deflection curve.

**Fig 10:**
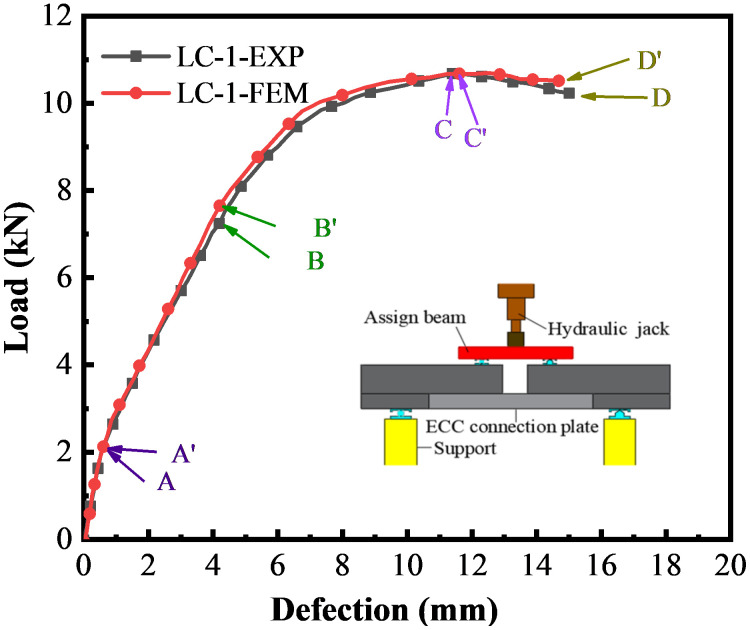
Comparison of the finite element model and the load-deflection curve obtained from the test.

Numerical simulations were performed for LC-1, and the results obtained from the finite element model were compared with the experimental data, as illustrated in Fig 10.

As shown in Fig 10, the finite element model demonstrates good agreement with the load-deflection curve obtained from the experimental test. The segment OA represents the elastic stage prior to cracking of the test beam. In the case of LC-1, cracking initiates at point A, corresponding to a cracking load of 2.1 kN. The segment AB corresponds to the post-cracking stage, during which the longitudinal tensile reinforcement yields at point B, with a yield load of 7.7 kN. At this stage, concrete cracking progresses, the height of the compression zone decreases, the neutral axis shifts upward, and the overall stiffness of LC-1 begins to degrade. The segment BD represents the failure stage, characterized by rapid crack propagation, a sharp upward movement of the neutral axis, a significant reduction in stiffness, and a substantial increase in deflection. During this stage, concrete crushing occurs, and the ultimate load at point D is recorded as 9.6 kN.

The stiffness predicted by the finite element model during the elastic phase is slightly higher than the experimental value. The primary reasons for this discrepancy include the following factors: a. The finite element model assumes idealized and strictly defined boundary conditions, whereas in the actual experiment, factors such as installation inaccuracies and support settlement make it difficult to replicate these conditions precisely. b. The finite element model typically neglects the slip behavior between rebar and concrete, while in reality, bond slip can lead to a reduction in structural stiffness. c. The constitutive relationship of the rebar in the model commonly employs a bilinear stress-strain model, which does not fully account for the strain hardening behavior and failure characteristics during the loading process. As a result, the degradation of the test beam’s load-bearing capacity after steel yielding in the later loading stages is not accurately captured by the model. d. Additionally, material properties in the finite element model are usually idealized and homogenized, without considering real-world variations such as aggregate distribution inhomogeneity and the presence of initial micro-cracks in the concrete, all of which can influence the structural stiffness observed in the experiment.

The finite element model predicts an ultimate load of 10.6 kN, with a deviation of 2.7% compared to the experimental value obtained from the test beam. This discrepancy may be attributed to several factors, including the simplification of the finite element model, the deviation between the actual mechanical behavior of the concrete and rebar and their idealized constitutive relationships, variations in the loading rate during the experiment, and limitations in the accuracy of the measuring instruments. Considering the inherent heterogeneity of concrete materials and the complexity of the experimental conditions, the observed error falls within an acceptable range.

Overall, the finite element model demonstrates good agreement with the experimental results in terms of key mechanical indicators, including stiffness variation trends and ultimate load capacity. This indicates that the model possesses a high level of predictive accuracy. Despite minor discrepancies, the errors remain within an acceptable range, and the finite element model is capable of effectively capturing the primary mechanical behavior of the specimen throughout the loading process.

#### 3.1.2. Failure state.

**Fig 11:**
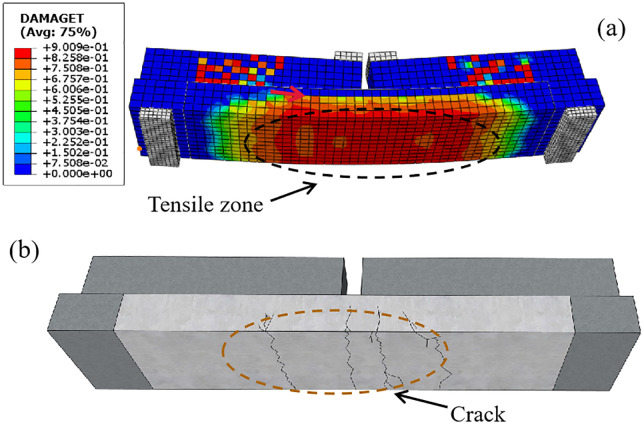
Crack distribution: **(a)**LC-1-FEM; **(b)**LC-1-EXP.

Finite element models are capable of accurately simulating the damage state and crack distribution of RC beams. The concrete plastic damage model incorporates the concept of damage, enabling the description of concrete behavior under cyclic loading conditions. The DAMAGET contour plot is an effective tool for visualizing crack development. The finite element simulation results of LC-1, along with the experimentally observed damage and crack distribution, are presented in Fig 11.

Fig 11(a) presents the damage distribution of LC-1 at failure as simulated by the finite element model, while Fig 11(b) illustrates the crack pattern obtained from the experimental test. A high degree of consistency can be clearly observed between the damage distribution predicted by the finite element model and the actual crack development in the test beam.

The failure mechanism of the ECP is primarily attributed to the excessive width of one or two dominant cracks. During the loading process, micro-cracks initially develop within the material, and their number increases with the applied load. According to the damage distribution map from the finite element model, the region with concentrated damage aligns closely with the location of the main crack observed in the experimental beam. Furthermore, the extent of micro-damage in the model is approximately equivalent to the micro-crack propagation zone in the experiment. This demonstrates that the finite element model is capable of not only accurately capturing the key factors contributing to ECP failure, but also effectively simulating the entire failure process—from the initiation of micro-cracks, their progression, to the formation of major cracks and eventual structural collapse.

Although the stiffness of the finite element model in the elastic stage is slightly higher than the experimental value, and there is a 2.7% discrepancy in the ultimate load, these differences remain within an acceptable range. The accuracy of the finite element model was further validated by comparing the damage distribution and crack patterns at the failure stage of LC-1 from the perspective of structural failure morphology and crack propagation behavior. In summary, the structural damage and crack distribution of LC-1 show a high degree of consistency between the finite element model and the experimental beam. This observation corroborates the previously demonstrated good agreement in mechanical performance indicators such as stiffness and ultimate load, thereby fully confirming the high reliability of the finite element model in capturing the mechanical behavior and failure characteristics of the ECP. As such, the model provides a solid foundation for further investigation into the mechanical properties of the ECP.

### 3.2. Parameter studies

To systematically investigate the influence of various parameters on the performance of the ECP, parametric analyses were carried out based on the validated finite element model, focusing on four key parameters: ECP thickness, longitudinal tensile rebar ratio, concrete strength, and ECC strength. The detailed specifications of the specimens corresponding to each parameter group are summarized in Table 5. For clarity and consistency in subsequent analysis and presentation, all specimens have been assigned uniform identification codes. The naming convention is as follows: “L” denotes the baseline ECP specimen; “EH” indicates the thickness variation group of the ECP; “S” represents the rebar ratio variation group; “C” corresponds to the concrete strength variation group; and “E” refers to the ECC strength variation group.

**Table 5:** Specimen Parameter Information.

No.	ECC strength (MPa)	Longitudinal tensile rebar diameter (mm)	Concrete strength (MPa)	Thickness of ECP (mm)
FLC-1	–	6	30	–
LEH-1	35	6	30	60
LEH-2	35	6	30	62
LEH-3	35	6	30	64
LEH-4	35	6	30	66
LEH-5	35	6	30	68
LS-1	35	6	30	60
LS-2	35	–	30	60
LS-3	35	8	30	60
LS-4	35	9	30	30
LS-5	35	10	30	30
LC-1	35	6	30	60
LC-2	35	6	40	60
LC-3	35	6	50	60
LE-1	35	6	30	60
LE-2	40	6	30	60
LE-3	45	6	30	60

**Fig 12:**
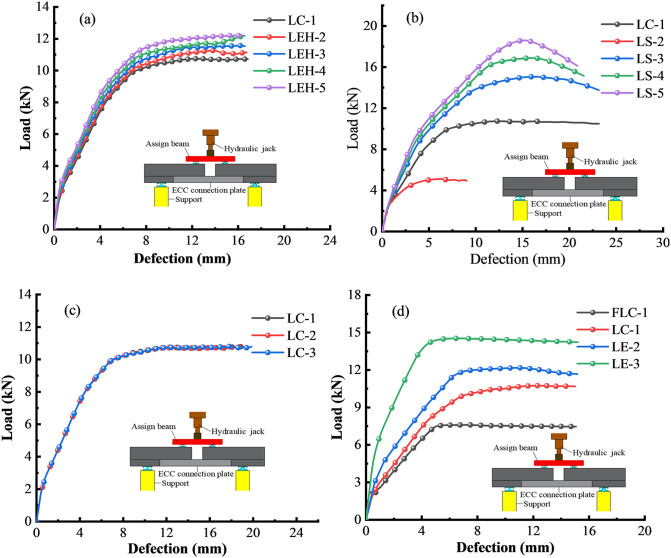
Load-deflection curves of ECP at different parameters: (a) thickness of ECP;(b) Rebar ratio; (c) Concrete strength; (d) ECC strength.

The load-deflection curves for each parameter group of specimens, obtained through the finite element model, are presented in Fig 12.

#### 3.2.1. Thickness of the ECP.

Through the analysis of how variations in the thickness of the ECP influence ECP performance, it is evident that ECC thickness has a significant impact on the load-bearing capacity of the connection. Therefore, a parametric study on ECC layer thickness is essential for achieving an optimized design. In this study, LC-1 was selected as the baseline model, and the ECC thickness was varied to 62 mm, 64 mm, 66 mm, and 68 mm, respectively. The corresponding yield load and ultimate load of the ECP under each thickness were then compared. As illustrated in Fig 12(a), the load-deflection curves of all test groups exhibited a high degree of similarity. During the elastic loading phase, the curves largely overlapped, indicating that the ECC had not yet been fully engaged. Upon entering the elastoplastic stage, the ECC began to exert its restraining effect, and the load-bearing capacity of the ECP increased progressively with the increase in ECC layer thickness.

**Fig 13:**
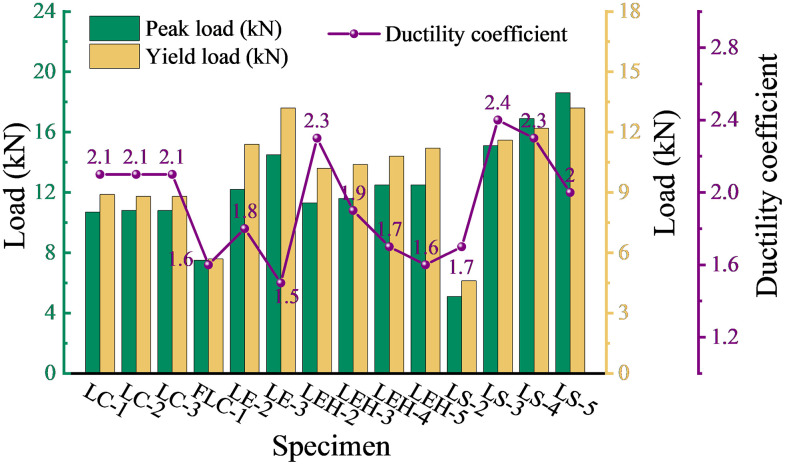
Ultimate load, yield load and ductility coefficient of ECP.

As illustrated in Fig 13, the ultimate loads for LC-1, LEH-2, LEH-3, LEH-4, and LEH-5 are 10.6 kN, 11.3 kN, 11.6 kN, 12.5 kN, and 12.5 kN, respectively. The corresponding yield loads are 7.7 kN, 10.2 kN, 10.4 kN, 10.8 kN, and 11.2 kN, respectively. Compared with LC-1, the bearing capacities of LEH-2, LEH-3, LEH-4, and LEH-5 increased by 5.6%, 8.4%, 16.8%, and 17%, respectively, while the yield loads increased by 32.5%, 35.1%, 40.3%, and 45.5%, respectively. This improvement can be attributed to the fact that as the thickness of the ECP increases, the compression zone becomes strengthened, the neutral axis shifts upward, and the ECC exhibits higher compressive strength and ultimate compressive strain compared to the concrete used in LC-1, thereby effectively enhancing the load-bearing capacity of the ECP. Additionally, with increasing ECP thickness, the ductility of the ECP undergoes a noticeable change. The ductility coefficients for LC-1, LEH-2, LEH-3, LEH-4, and LEH-5 are 2.1, 1.9, 2.3, 3.1, and 3.2, respectively, indicating an initial decrease followed by a gradual increase in ductility.

The ECP can enhance the stiffness and fatigue resistance of the bridge deck, thereby alleviating stress concentration in the steel plate, which is consistent with the findings reported by Das et al. [33]. However, it should be noted that although the ultimate bearing capacity increases with the thickness of the ECC layer, the improvement is relatively marginal. Considering the high flexural and tensile strength of ECC, its thickness can be appropriately reduced in practical engineering applications to reduce material costs without compromising structural performance.

#### 3.2.2. Rebar ratio.

Through the analysis of how variations in the longitudinal tensile rebar ratio affect ECP performance, it is evident that the longitudinal rebar significantly influences the load-bearing capacity and crack resistance of the ECP. As illustrated in Fig 13, compared with LC-1, the ultimate bearing capacities of LS-3, LS-4, and LS-5 increased by 42.5%, 58.5%, and 75.5%, respectively, while their yield loads increased by 50.6%, 58.4%, and 71.4%, respectively. This demonstrates a clear positive correlation between the reinforcement ratio of longitudinal tensile rebar and the structural capacity of the ECP. In contrast, LS-2, which lacks longitudinal rebar, exhibits a substantial reduction in both yield and ultimate loads. Specifically, compared to LC-1, the yield load of LS-2 decreased by 42.5%, and the ultimate load decreased by 52.3%. These results indicate that the presence of longitudinal tensile rebar plays a critical role in enhancing the mechanical performance of the ECP.

As shown in the load-deflection curve presented in Fig 12(b), the loading process of the ECP can be broadly categorized into three distinct stages. The first stage is the crack-free stage, during which the ECP remains uncracked and behaves in a linear elastic manner. The load-deflection curves of all test specimens exhibit similar linear trends, indicating that the longitudinal tensile rebar significantly influences the cracking load and initial stiffness of the structure. The second stage is characterized by crack propagation. During this phase, micro-cracks begin to develop, leading to a slight reduction in stiffness. The number of cracks increases rapidly, while their width grows at a slower rate. The third stage is the rebar yielding stage. Once the tensile rebar yield, the bending moment and number of cracks remain relatively stable, whereas the mid-span deflection and crack width increase significantly. After the ECP reaches its ultimate load, the descending branch of the load-deflection curve exhibits notable differences. The descending branch becomes steeper for specimens with higher rebar ratios, indicating a reduced deformation capacity at failure. As the rebar ratio increases, the deflection at failure gradually decreases. Specifically, the ductility coefficients of LC-1, LS-2, LS-3, LS-4, and LS-5 are 2.1, 1.7, 2.4, 2.3, and 2.0, respectively, corresponding to longitudinal tensile rebar ratios of 1.1%, 0%, 2%, 2.4%, and 3%. Compared to LC-1, the ductility coefficient of LS-2 decreased by 23.5%, while those of LS-3, LS-4, and LS-5 increased by 14.3%, 9.5%, and 4.7%, respectively. Notably, the ductility coefficient of LS-5 slightly decreased by 5% after reaching its peak. These findings suggest that the ductility of the ECP initially increases gradually with the rebar ratio, but at a diminishing rate, and subsequently begins to decline. At higher rebar ratios, the ECP exhibits more brittle behavior. Therefore, it can be concluded that when the rebar ratio falls within the range of 2% to 3%, its influence on both the load-bearing capacity and ductility of the ECP becomes more pronounced.

#### 3.2.3. Strength of concrete.

Through the analysis of how variations in concrete strength affect the performance of the ECP, it can be observed from Fig 12(c) that at the initial stage of loading, the ECP remains in the elastic deformation phase, with the load-deflection curve exhibiting an approximately linear distribution. When the load reaches 2.3 kN, the ECP begins to transition into the plastic phase. At this point, initial tensile cracks appear in the concrete, and as the load continues to increase, the longitudinal tensile rebar gradually yields, ultimately leading to structural failure.

From the perspective of curve morphology, the load-deflection curves of all test beams are highly consistent, with similar yield loads and ultimate bearing capacities. Further analysis, in conjunction with the data presented in Fig 13, indicates that the ductility of the ECP does not exhibit significant variation with increasing concrete strength. This phenomenon can be primarily attributed to the fact that, in the ECP structure, the tensile zone is predominantly governed by the ECC, which carries the tensile stresses and controls crack propagation. Meanwhile, the concrete mainly functions within the compression zone. When its strength falls within a reasonable range, the concrete in the compression zone does not act as the dominant factor influencing structural behavior. Consequently, variations in concrete strength have a relatively minor effect on the overall load-bearing capacity and ductility of the ECP.

#### 3.2.4. ECC strength.

Through the analysis of how variations in ECC strength influence the performance of the ECP, it can be observed from Fig 12(d) that the load-deflection curves exhibit linear behavior at the initial stage of loading, with generally consistent trends. Before cracking occurs, a higher ECC strength corresponds to a greater elastic modulus, which in turn increases the section modulus of the ECP. This leads to an elevated cracking load and a steeper slope of the load-deflection curve during the elastic stage. This indicates that the initial stiffness of the ECP is enhanced. Upon entering the ultimate limit state, the ECP undergoes strain hardening. At this stage, ECC with higher tensile strength can sustain greater stress under the ultimate load, thereby significantly improving the load-bearing capacity of the ECP. Once the ultimate limit state is reached, the load-deflection curve begins to descend.

As shown in Fig 13, with an increase in ECC strength, the ultimate loads of FLC-1, LC-1, LE-2, and LE-3 are 7.5 kN, 10.7 kN, 12.2 kN, and 14.5 kN, respectively, with corresponding deflections of 5.3 mm, 11.8 mm, 10.9 mm, and 5.6 mm. The respective yield loads are 5.7 kN, 7.7 kN, 11.4 kN, and 13.2 kN, with corresponding deflections of 3.4 mm, 5.6 mm, 6.2 mm, and 3.7 mm. Compared to LC-1, the ultimate load of FLC-1 is reduced by 50.4%, the yield load by 26%, and the ductility coefficient by 27.3%. This significant reduction is primarily due to the use of ordinary concrete instead of ECC in FLC-1, which exhibits inferior tensile performance and is unable to maintain a high load-bearing capacity after cracking, thereby leading to a decline in overall mechanical performance. In contrast, LE-2 and LE-3, which incorporate higher-strength ECC, demonstrate an increase in ultimate bearing capacity by 14.0% and 35.5%, respectively, and an increase in yield load by 12.9% and 30.7%, respectively, compared to LC-1. However, their ductility coefficients decrease by 14.3% and 28.6%, respectively. This is attributed to the increased brittleness and reduced deformability associated with higher ECC strength. Although the material can withstand greater loads, its plastic deformation capacity diminishes after reaching the ultimate state, resulting in reduced ductility. As discussed above, increasing the strength of ECC can effectively enhance the load-bearing capacity of the ECP. However, this improvement comes at the cost of reduced ductility due to the increased brittleness of the material.

### 3.3. Strain

The finite element model was utilized to extract the strain distribution curve along the height of the mid-span section of the ECP and the relationship curve between rebar strain and applied load. These results serve as a theoretical foundation for the subsequent calculation of the ECP’s load-bearing capacity.

#### 3.3.1. Strain distribution across sections.

**Fig 14:**
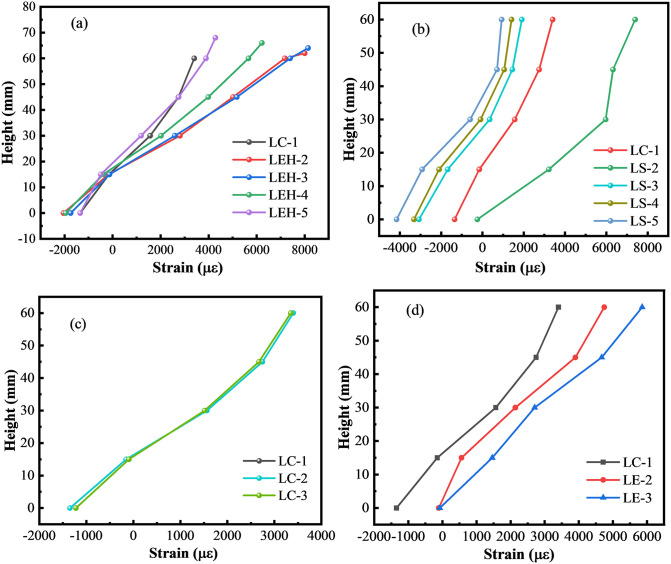
Strain distribution curves of the ECP at different parameters along the height of the cross-section at mid-span: **(a)** Thickness of the ECP; **(b)** Rebar ratio; **(c)** Concrete strength; **(d)** ECC strength.

Fig 14 presents the strain distribution curves of the mid-span section of the ECP. During the loading process, the strain in the ECP gradually increases with the applied load, and the cross-section exhibits a distribution pattern characterized by compression at the upper edge and tension at the lower edge. The maximum strain is observed at the lower edge of the cross-section.

Specifically, Fig 14(a) illustrates that under the same applied load, the strain in the ECP does not monotonically increase with the thickness of the ECC layer. Compared with LC-1, the maximum tensile strains of LEH-2, LEH-3, LEH-4, and LEH-5 increased by 140%, 140%, 82.8%, and 25.9%, respectively. Notably, LEH-2 and LEH-3 exhibit identical maximum strain values at the same height. Fig 14(b) demonstrates the influence of the rebar ratio on ECP strain. The maximum tensile strains of LS-3, LS-4, and LS-5 decreased by 43.9%, 58.6%, and 72.6%, respectively, compared to LC-1. This indicates that the maximum tensile strain of the ECP decreases as the rebar ratio increases. This phenomenon can be attributed to the fact that a higher rebar ratio enhances the tensile and flexural stiffness of the ECP, thereby reducing both the tensile strain and tensile stress in the structure. In contrast, LS-2 experienced greater deformation and failure due to the absence of longitudinal tensile rebar. As shown in Fig 14(c), variations in concrete strength have no significant effect on the strain distribution of the ECP. Fig 14(d) presents the relationship between the elastic modulus of ECC and the strain of the ECP. A higher elastic modulus of ECC corresponds to a higher strain in the ECP. The maximum tensile strains of LE-2 and LE-3 were 39.5% and 72.2% greater, respectively, than that of LC-1. Under the same stress conditions, a higher strain indicates a higher strength of the ECC, meaning that the ECP can sustain greater deformation without failure [45].

#### 3.3.2. Load-strain curves of rebar.

**Fig 15:**
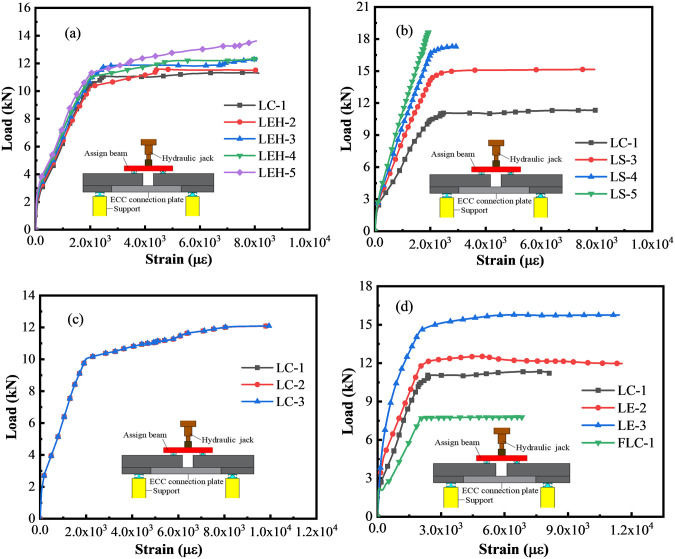
Load-strain curves of the rebar at different parameters: **(a)** Thickness of the ECP; **(b)** Rebar ratio; **(c)** Concrete strength; **(d)** ECC strength.

Fig 15 presents the load-strain curves of the tensile rebar in the ECP. The increase in tensile strain of these rebar reflects the development status of vertical cracks within the constant bending moment region of the ECP. Overall, compared to LC-1, increasing the thickness of the ECC layer, the rebar ratio, and the ECC strength all result in higher rebar strains. In contrast, variations in concrete strength do not significantly affect the load-strain behavior of the rebar.

Specifically, when the load increases from 0 to 2.1 kN, the load on the rebar is gradually transferred to the ECC. At this stage, both slip and strain values remain relatively low, and the load-strain curve exhibits a steep slope, indicating a nearly linear relationship between load and strain. Prior to reaching the yield load, the bond between the rebar and ECC gradually deteriorates, leading to a reduction in the slope of the load-strain curve and the initiation of internal cracks extending toward the surface of the ECP. As the load continues to increase, crack propagation progresses, and the strain in the rebar rises accordingly. Fig 15(a) illustrates that the ECP and the rebar exhibit coordinated deformation characteristics. Under the same loading conditions, a greater thickness of the ECC layer corresponds to a lower strain in the rebar, suggesting that the application of ECC in the tensile zone effectively enhances the utilization of the rebar’s strength. However, it should be noted that an excessively thick ECC layer may lead to brittle failure of the ECP. Fig 15(b) demonstrates that as the diameter of the rebar increases from 6 mm to 10 mm, the strain value decreases. This is attributed to the fact that ECP specimens with a higher rebar ratio tend to exhibit brittle failure characteristics, which result in a reduction in structural ductility. Fig 15(c) indicates that variations in concrete strength have no significant influence on the strain of the rebar. As shown in Fig 15(d), the load-strain curve of the rebar in the ECP is significantly higher than that in FLC-1. This is due to the relatively low elastic modulus of ECC, which allows for greater tensile and compressive deformation and provides excellent durability and fatigue resistance. These properties enable ECC to better fulfill the functional requirements of expansion joints. Furthermore, the load-strain curve values of the rebar in LE-2 and LE-3 are higher than those in LC-1, indicating that increasing the strength of ECC can significantly improve both the load-bearing capacity and deformation capacity of the ECP. Under the same load, higher ECC strength enhances the bonding force between internal fibers and the matrix, effectively suppressing crack propagation and penetration, thereby delaying the structural failure of the ECP.

### 3.4. Flexural bearing capacity prediction

When conducting a theoretical analysis of the mechanical behavior of the ECP, the RC bridge deck and main beam are simplified into T-sections consisting of flanges (representing the bridge deck) and webs (representing the main beam). This simplification accounts for the combined contribution of the top slab and the main beam to the section’s bending moment of inertia, which reflects the actual structural behavior where different components work together under load. At the same time, considering the characteristics of a cracked section, the tensile strength of the concrete is neglected. This is because the tensile strength of concrete is relatively low, and its contribution to load-bearing capacity becomes negligible after cracking. This simplified approach aligns with the conventional engineering practice of disregarding the tensile resistance of concrete in the tension zone. Based on the above assumptions, the moment of resistance of the section can be calculated using Equations (4) and (5).


$$\begin{array}{c} M=\phi_{s}f_{y}A_{s}(d-\frac{a}{\text{2}}) \end{array}$$ (4)

$$\begin{array}{c} a=\frac{\phi_{s}f_{y}A_{s}(d-\frac{a}{\text{2}})}{\alpha_{\text{1}}\phi_{c}\overset{\prime }{\underset{c}{f}}b_{e}} \end{array}$$ (5)

Where, $\phi_{s}$ is the reduction coefficient of steel, $f_{y}$ and $A_{s}$ are respectively the yield strength and cross-sectional area of the rebar, *d* is the effective section height, *a* is the depth of the rectangular stress block calculated according to Equation (5), $\phi_{c}$ and $\overset{\prime }{\underset{c}{f}}$ are respectively the strength reduction coefficient and compressive strength of the concrete, and $b_{e}$ is the effective flange width.

**Fig 16:**
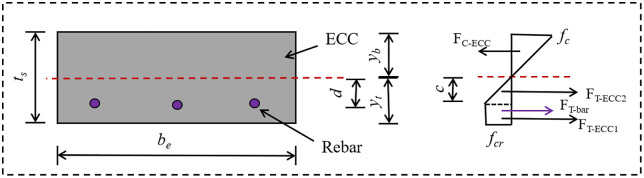
Schematic diagram of the theoretical analysis of ECP.


Assume that the ECP generates a moment by rotating at the ends of adjacent span beams. Therefore, the ECP is analyzed as a flexural member. The ECC tensile components *F*_*T-ECC1*_ and *F*_*T-ECC2*_ for rectangular and triangular stress distributions were considered respectively, as shown in Fig 16. Therefore, the sum of each component force is considered to determine the flexural bearing capacity: the rebar tension *(F*_*T-bar*_*)*, the ECC tension (*F*_*T-ECC1*_ and *F*_*T-ECC2*_), and the ECC pressure (F_C-ECC_). The tensile component of ECC can be determined by *c* in Fig 16. It should be noted that this formulation is applicable to ECC materials exhibiting strain-hardening behavior with stable multiple cracking, while the specific value of the tensile parameters should be calibrated according to the mechanical properties of different ECC types [46]. Total flexural bearing capacity $M_{\text{r-ls}}$ is determined by Equation (6).

$$\begin{array}{c} M_{r-ls}=F_{T-ECC1}\times \left(\frac{y_{t}-c}{\text{2}}+c\right)+F_{T-ECC2}\times \frac{\text{2}}{\text{3}}c+F_{T-bar}\times (y_{t}-d)+F_{C-ECC}\times \frac{\text{2}}{\text{3}}y_{b} \end{array}$$ (6)

Where, *b*_*e*_ is the effective width of the ECP, *t*_*s*_ is the total thickness of the ECP, *d* is the distance from the top surface to the center of the reinforcement, *y*_*b*_ and *y*_*t*_ are the distances from the central axis to the top and bottom surfaces respectively, *f*_*cr*_ is the crack strength of the ECC, and *f*_*c*_ is the compressive strength of the concrete.

Table 6 presents the flexural load-bearing capacities of the five specimens obtained through both analytical calculations and finite element modeling. The relative errors for the five specimens are −5.0%, −5.0%, −1.2%, −2.9%, and 6.8%, respectively. The calculated average error is −1.46%, with a coefficient of variation of 3.345. According to the data, the discrepancy between the theoretical model and the finite element model remains within 5%, indicating a high degree of consistency between the two methods and further validating the accuracy of the theoretical model.

**Table 6:** Comparison of calculation results between the theoretical model and the finite element model.

No.	M_FE_ (kN.m)	M_r-ls_ (kN.m)	(M_r-ls_-M_FE_)/M_FE_
LC-1	2.00	1.90	−0.050
LC-2	2.00	1.90	−0.050
LEH-2	4.85	4.79	−0.012
LS-5	5.80	5.63	−0.029
LE-2	5.45	5.82	0.068
Average(%)			−1.46
Coefficient of Variation			3.345

## 4. Conclusions

Based on the numerical simulation of the ECP and an in-depth investigation into its mechanical behavior, the following conclusions have been drawn:

(1) Utilizing ECP as a connecting plate in RC seamless bridges effectively enhances their flexural load-bearing capacity, ductility, and deformation capacity. Compared to FLC-1, the yield load, ultimate load, and ductility coefficient of LC-1 increased by 41.3%, 35.1%, and 31.3%, respectively, indicating that the incorporation of ECC provides a pronounced improvement in structural performance and deformation capacity.
(2) Increasing the thickness of the ECC layer contributes to improved flexural bearing capacity and ductility of the ECP. Compared to LC-1, the load-bearing capacities of LEH-2, LEH-3, LEH-4, and LEH-5 increased by 5.6%, 8.4%, 16.8%, and 17.0%, respectively. The results suggest that an appropriate increase in ECC thickness is an effective design parameter for enhancing flexural performance.
(3) Enhancing the rebar ratio significantly increases the ultimate load of the ECP; however, the maximum tensile strain decreases with increasing rebar ratio, indicating a trade-off between strength and deformation capacity. Compared to LC-1, the ultimate bearing capacities of LS-3, LS-4, and LS-5 increased by 42.5%, 58.5%, and 75.5%, respectively.
(4) Elevating the ECC strength markedly improves the flexural capacity of the ECP. Compared with LC-1, the ultimate bearing capacities of LE-2 and LE-3 increased by 14.0% and 35.5%, respectively, demonstrating the sensitivity of ECP performance to ECC material properties.
(5) The theoretical model proposed in this study exhibits high predictive accuracy, with errors within 5%, an average error of –1.46%, and a coefficient of variation of 3.345, confirming its applicability for predicting the flexural capacity of ECPs in seamless bridge applications.
(6) Further research is required to validate the proposed model under varying interfacial conditions and long-term effects, and to explore hybrid composite systems incorporating ECC and other sustainable materials.
